# Modeling Gene Regulation in Liver Hepatocellular Carcinoma with Random Forests

**DOI:** 10.1155/2016/1035945

**Published:** 2016-10-12

**Authors:** Hilal Kazan

**Affiliations:** Department of Computer Engineering, Faculty of Engineering, Antalya International University, Antalya, Turkey

## Abstract

Liver hepatocellular carcinoma (HCC) remains a leading cause of cancer-related death. Poor understanding of the mechanisms underlying HCC prevents early detection and leads to high mortality. We developed a random forest model that incorporates copy-number variation, DNA methylation, transcription factor, and microRNA binding information as features to predict gene expression in HCC. Our model achieved a highly significant correlation between predicted and measured expression of held-out genes. Furthermore, we identified potential regulators of gene expression in HCC. Many of these regulators have been previously found to be associated with cancer and are differentially expressed in HCC. We also evaluated our predicted target sets for these regulators by making comparison with experimental results. Lastly, we found that the transcription factor E2F6, one of the candidate regulators inferred by our model, is predictive of survival rate in HCC. Results of this study will provide directions for future prospective studies in HCC.

## 1. Introduction

Liver hepatocellular carcinoma (HCC) is the third leading cause of cancer-related mortality in the world. Furthermore, HCC is one of the few cancer types whose incidence and mortality rates are increasing worldwide. Despite this, HCC is a relatively understudied cancer that lacks biomarkers for prognosis and the regulatory factors involved in carcinogenesis remain uncharacterized [1]. As such, identification of the regulatory pathways leading to cancer development is critical for the development of efficient therapeutic strategies.

Cancer development is commonly associated with altered gene expression. Changes in gene expression can be associated with copy-number variation (CNV) as well as with more complex patterns of dysregulation in gene expression control. Gene expression regulation can be grouped as transcriptional and posttranscriptional regulation. Transcriptional regulation is mainly controlled by binding of transcription factors (TFs) to gene promoters to control transcription rate. TFs can either activate or repress gene expression. The methylation level of the promoter regions also affects the transcription rate. Gene expression can also be controlled after transcription. MicroRNAs (miRNAs) are an important class of small RNAs acting in posttranscriptional regulation. miRNAs bind to their sites in 3′ untranslated regions (UTRs) of target genes and repress their expression. Aberrations in the functioning of TFs and miRNAs can lead to major changes in gene expression.

The Cancer Genome Atlas (TCGA) has been profiling hundreds of tumor samples at multiple molecular and regulatory layers including measurements of gene copy number, DNA methylations, and mRNA and miRNA expression [2]. Encyclopedia of DNA Elements (ENCODE) is another large-scale genomic project that aims to map the genome-wide binding sites of TFs using ChIP-seq [3]. The availability of these rich datasets provides opportunities to develop integrative computational models to understand molecular mechanisms of carcinogenesis.

Recently, Setty et al. proposed a lasso regularized regression model that incorporates copy-number variation, DNA methylation levels, TF, and miRNA-mediated regulatory effects to predict differential gene expression in glioblastoma (GBM) [4]. Their model identified regulators of GBM whose activities are correlated with patient survival rate. Jacobsen et al. utilized TCGA data to identify recurrent patterns of miRNA-mRNA associations across 11 cancer types [5]. In this model, copy-number variation and DNA methylation level are included but TF-based regulation is ignored. Ignoring TF-based regulation might have led to overfitting and identification of spurious miRNA-mRNA associations. RACER is a statistical model that takes into account both transcriptional and posttranscriptional regulation as well as copy-number variation and DNA methylation within a two-stage regression framework to infer the candidate regulators in acute myeloid leukemia [6]. Recently, Li et al. predicted differential gene expression in lung cancer by using a comprehensive feature set that represents epigenetic alterations but ignoring TF- and miRNA-based regulation [7].

Here, we propose to predict gene expression in liver hepatocellular carcinoma with a statistical model that incorporates copy-number variation and DNA methylation as well as the regulatory effects of TFs and miRNAs. Unlike many of the previous studies that use linear regression, we used random forests to represent the potential nonlinear relationships between the regulatory factors and gene expression. Random forests are also suitable for this problem as they can deal with large number of correlated features and higher order interactions. Furthermore, rather than predicting expression of genes sample by sample independently, we aimed to predict the variation of expression of a single gene across the tumor samples to better capture the target relationships of TFs and miRNAs. We achieved an average Spearman correlation of 0.65 and determined the key regulators controlling gene expression. Additionally, we inferred the target gene sets of top regulators to better understand their activities and found that one of these regulators, E2F6, is predictive of survival in HCC.

## 2. Methods

### 2.1. Data Collection

We used TCGA data portal to compile the liver hepatocellular carcinoma datasets on mRNA expression and miRNA expression. We downloaded RNA-seq Level 3 datasets that correspond to mRNA expression. Similarly, we downloaded Illumina HighSeq and Illumina GA datasets for miRNA expression. We applied log_2_ transformation of RNA-seq read counts. Additionally, we downloaded DNA copy-number variation (GISTIC2-processed) and DNA methylation levels from Firehose database (analyses: 01-11-2015) [8]. We determined the methylation probe that shows the largest negative correlation between “Beta-value” and mRNA expression across all the samples and used its value as the methylation level.

We defined the set of expressed TFs in liver by taking the union of the expressed proteins in Human Proteome Map [9] and Human Protein Atlas [10] using the adult liver and liver cancer cells, respectively. We downloaded the survival times of liver hepatocellular carcinoma patients from UCSC cancer genomics browser.

### 2.2. Differential Expression Analysis with* edgeR*

We used an existing method called* edgeR* to determine the genes that are differentially expressed in cancer [11]. We prepared an input matrix of RNA-seq read counts where rows are genes and columns are paired tumor-normal samples. We discarded the mRNAs that have less than 4 reads in more than 70% of the samples.* edgeR* finds the genes that are differentially expressed in tumor cells compared to normal cells, adjusting for baseline differences between the patients. First, we used the function* calcNormFactors* to adjust for varying sequencing depth and RNA composition effects across samples. This function gives scaling factors which are then used to calculate effective library sizes. Effective library size replaces the original library size in all subsequent analyses.* edgeR* models the RNA-seq read counts with negative binomial distribution. The next step is to calculate the dispersion estimates by fitting negative binomial models. Lastly, differential expression is determined using the generalized linear model (GLM) likelihood ratio test. We defined those genes with absolute log fold changes greater than 1 and FDR-corrected *p* values less than 0.05 to be differentially expressed. This selection resulted in 1220 upregulated genes and 2296 downregulated genes. We repeated the same analysis for miRNAs that have more than 1 read count in more than 70% of the samples.

### 2.3. Mapping Binding Sites of Transcription Factors and MicroRNAs

We defined the promoter regions as the 2000 nt region upstream or downstream of the transcription start sites based on Refseq annotation. We downloaded the position frequency matrices (PFMs) of 382 TFs from JASPAR database (JASPAR 2016 release [12]). After intersecting with expressed TFs in liver, there remained 241 TFs. Next, we scanned the promoter regions with these PFMs using the FIMO tool from the MEME-suite [13]. We kept the sites with *p* values less than 1*e* − 4. Lastly, we formed a binary matrix of TFs and genes where an entry of 1 indicates that the corresponding TF has at least one binding site for the corresponding gene. As an alternative, we downloaded ChIP-seq datasets of 59 TFs in HepG2 cells from ENCODE project. For each TF and gene, the corresponding element of the matrix is 1 if the gene contains a ChIP-seq peak for that TF. Otherwise, the element is labeled with 0.

We defined the miRNA target sites as conserved targets from the recent TargetScan release (v7 [14]). This release of TargetScan defines the human 3′UTRs by extending Gencode annotations with 3′UTR isoforms and alternative polyadenylation. To define the features that correspond to miRNAs, we formed a binary matrix similar to the matrix we formed for TFs. After intersection with the set of miRNAs for which we have miRNA-seq data, we were left with 360 miRNAs.

In the end, we found that 360 of the 373 samples have CNV, methylation, and miRNA-seq data. Also, we filtered out the genes that have missing CNV or methylation data and those genes with no TF or miRNA site. Our final dataset consists of the expression data of 2689 genes across 360 samples.

### 2.4. Random Forest

Random forest is an ensemble learning algorithm that consists of a collection of decision trees [15]. Each tree is fit independently with a set of bootstrapped samples. In addition to the randomness involved with bootstrapping, each node of a tree is split using the best among a subset of variables randomly chosen at that node. In particular, at each node, *n* candidate variables out of *N* total number of variables are randomly selected. The final variable to be used for splitting is determined based on a certain cost function. Outputs from individual trees are weighted to obtain the final outcome. One advantage of random forest is the ability to estimate error rate during training. Namely, at each bootstrap iteration, the tree that is trained with the bootstrap sample is tested with the samples that are not in the bootstrap sample. This test set is also called the “out-of-bag” or OOB data. After learning all the trees, predictions are aggregated to calculate the OOB estimate of error rate. Additionally, variable importance values are estimated by calculating the change in mean squared error in OOB data when each predictor variable is permuted.

We used the R package* randomForest* to run our model. We predicted the expression variation of a single gene across the samples. In addition to CNV and methylation level, we included TF and miRNA-mediated regulation by multiplying the expression of TFs or miRNAs for each sample with the binary binding information of TFs and miRNAs for that gene. When predicting the expression variation of a gene, the expression of a TF or miRNA is included as a feature if the gene contains at least one binding site for that TF or miRNA.

We chose the parameters* mtry* (the number of variables that is selected at each node) and* ntrees* (number of trees) using the* tuneRF* function. In particular, we ran* tuneRF* with a range of* ntree* values between 60 and 140. The best accuracy was observed with* mtry* = 70 and* ntree* = 100. Therefore, we ran our subsequent experiments with this parameter setting.

## 3. Results

### 3.1. Evaluation of the Random Forest Regression Model

**Figure 1:**
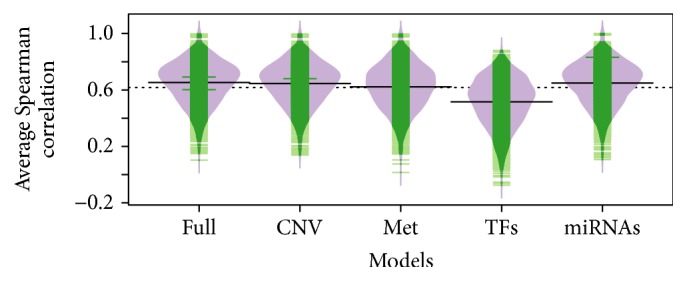
Comparison of the random forest models that exclude one type of feature at a time.

We used the random forest model to predict the expression variation of differentially expressed genes across the tumor samples. We used the built-in error estimation procedure in the random forest model and calculated the Spearman correlation of predicted gene expression with measured gene expression in the held-out samples (i.e., OOB data). We achieved an average correlation of 0.65 with the full model. We repeated the same evaluation by excluding each feature class one by one.  Figure 1 compares the performance of the full model with reduced models using a beanplot. Excluding TFs resulted in the largest decrease of performance (avg. correlation: 0.52). This confirms the critical roles of TFs in regulating gene expression. We found that the second most important factor type was methylation leading to an average correlation of 0.62. Exclusion of CNV and miRNAs showed relatively small but significant effect (avg. correlations are 0.645 and 0.648, resp.). The difference between full and reduced models was significant for all the cases (Wilcoxon sign-rank test *p* value < 2.2*e* − 16). We also tried replacing the TF features with ENCODE-based definition of TF binding sites. However, this resulted in lower performance possibly due to the smaller number of included TFs (avg. correlation: 0.54).

### 3.2. Identification of Key Regulators

**Figure 2:**
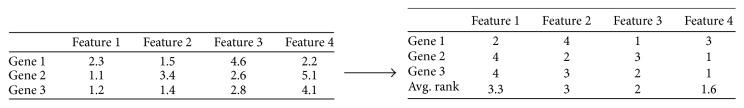
A toy example illustrating the calculation of ranks from importance values.

We utilized the variable importance measures estimated by random forest model to identify the most important features that predict gene expression in our model. Namely, the variable importance value of a feature corresponds to the change in mean squared error when that feature is permuted. For each gene, we obtained the importance values of all the features from the random forest model. Then, for each gene, we calculated the ranks of these importance values so that a smaller rank corresponds to a more predictive feature. Lastly, we formed a matrix where each row is a gene, each column is a feature, and entries correspond to the ranks of the importance values. For each feature, we averaged the ranks across all the genes to get an estimate of the importance of this feature for the set of all genes. Then, we sorted the features according to this average rank value in increasing order. A toy example is shown in Figure 2 where the table on the left contains the importance values predicted by the random forest model and the table on the right contains the ranks. In this toy example, Feature 4 would be identified as the most important regulator.

Table 1 shows the top features with lowest average ranks (i.e., highest importance). We observe that DNA methylation and CNV rank as the most important features confirming the significant effect of these factors on gene expression. The most important features that follow CNV and methylation are transcription factors. We also included the log fold changes and the associated FDR-corrected *p* values of our differential expression analysis in this table. We found that many of these transcription factors are significantly differentially expressed in cancer.

Our top ranking feature, GLIS3, belongs to the same family with another candidate regulator, GLIS2, and, together, they have been found to be associated with liver fibrosis [16]. Similarly, the next ranked regulator TCF3 has been found to regulate breast cancer cell differentiation and tumorigenicity [17]. Additionally, previous literature has revealed that TCF3 and TCF4 are overexpressed in rectal cancer and they control MYC expression in colorectal cells [18]. A previous study identified ZEB1 as a key promoter of metastasis in pancreatic and colorectal cancer cells [19]. E2F6 is another candidate regulator that belongs to a family of proteins that play critical roles in regulating cell proliferation and differentiation [20].

The top miRNA ranks 115th in our feature importance list. This result indicates that TFs have more widespread effects on gene expression as our ranking metric favors features with good importance values across many genes. This is in accordance with the fact that TFs can have dramatic effects on gene expression by changing the transcription rate, whereas miRNAs fine-tune the expression. The last rows of Table 1 include the top 3 miRNAs with lowest average ranks.

### 3.3. Target Gene Sets of Key Regulators

We utilized the matrix of importance ranks to also infer the targets of key regulators. We hypothesized that a regulator should appear as an important feature in predicting the expression of its target genes. As such, for each feature, we sorted the ranks across all the genes and defined the top of this list as predicted targets. To evaluate the accuracy of our predicted target sets we compiled the existing experimental data. In particular, we downloaded knockdown datasets for 59 TFs in lymphoblastoid cell line [21]. This dataset contains genome-wide measurements of gene expression when a TF is knocked down. For each TF, we sorted the genes according to *p* values calculated with likelihood ratio test comparing the knockdown samples to the controls. We then calculated the overlap between the top 500 targets inferred by our model with the 500 genes that have the lowest *p* values indicating a significant fold change between knockdown and control samples. We repeated the same analysis with previously published transfection data for miR-122 [14].  Table 2 lists the results of this overlap analysis. We found that on average 30% of our predicted target set overlaps with experimentally defined target set indicating the success of our model. In particular, E2F6 target set shows the largest overlap (165/500).

To further evaluate the predicted targets of candidate regulators, we performed a literature search to compile the known targets of some of the TFs in Table 2. Our literature search revealed that TCF3 interacts with the tumor suppressor CDKN2A in human fibroblast cells [22]. Indeed, CDKN2A is one of the TCF3 targets that we predicted with the random forest model and it also appears in the overlap target list for TCF3. Additionally, Ulgiati and Holers used EMSA method and identified that CR2 promoter activity is critically controlled by TCF3 [23]. Interestingly, CR2 appears in our predicted target list but not in the list of targets determined by the knockdown dataset. We observed a similar result for the TF ZEB1. A previous study showed that ZEB1 suppresses the expression of CDH13 (T-cadherin) in gallbladder cancer cells [24]. CDH13 is one of the predicted targets of ZEB1; however, it is not identified by the knockdown dataset. Xu et al. performed ChIP-chip analysis of E2F family in 5 different cell types (Ntera2, MCF10A, MCF7, HeLa, and GM06990) and identified the top ranking promoters (i.e. 434 targets) [25]. Of these, 8 genes are in the overlapping set of targets and 33 genes are in our set of predicted targets. Lastly, for REST, we downloaded the list of putative targets identified by ChIP-seq method in HepG2 cells [26] and found that 126 of them are also predicted by our model and 11 of them are in the overlapping set of targets. Altogether, these results further confirm the accuracy of our predicted targets.

We also checked whether the target sets of candidate regulators are enriched for gene ontology categories. We used the GOrilla tool [22] with our ranked target gene sets to identify the enriched GO biological process (BP) terms. The second and third columns of Table 2 show the enriched GO terms and *p* values corrected for multiple testing, respectively. We only included the top enriched GO terms in this table. This analysis revealed that activities of TCF3 and E2F6 are associated with cell cycle process indicating a potential function in cancer development.

### 3.4. Survival Analysis

**Figure 3:**
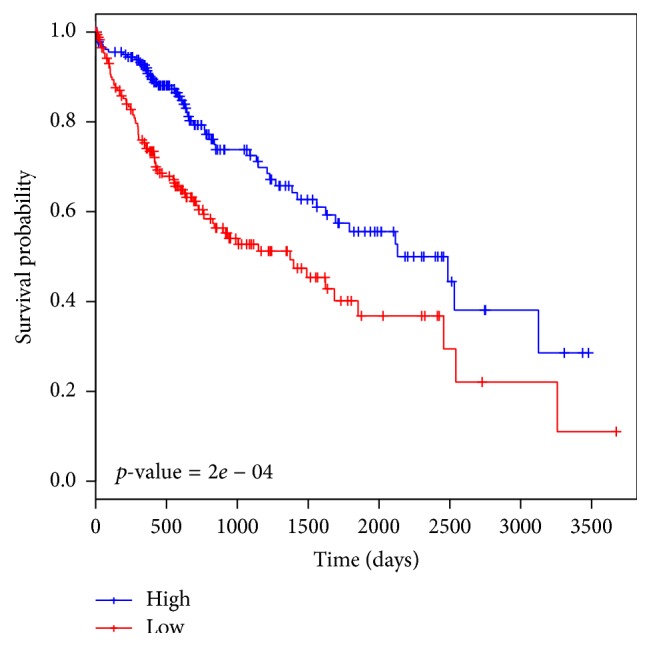
E2F6 expression is predictive of survival rate in HCC patients.

We used the R package* survival* to perform Kaplan-Meier analysis. We chose the median expression value (across the tumor samples) as the cutoff. This analysis revealed that the expression of E2F6 predicts the differences in survival time of HCC patients (log-rank test *p* value 2*e* − 04) (Figure 3). This result is in line with our previous finding that E2F6 is associated with cell cycle control. Further experimental studies will be instrumental in characterizing the molecular mechanisms behind the association of E2F6 with survival in HCC.

## 4. Conclusion

Understanding the gene regulatory mechanisms that lead to aberrant gene expression in cancer is critical for the identification of biomarkers and therapies. Here, we have developed a random forest model that integrates various genome-wide measurements including CNV, DNA methylation, and expression of TFs and miRNAs coupled with their binding information. By excluding each feature type at a time, we found that TFs show the greatest decrease in performance when excluded. This is followed by CNV, methylation, and miRNAs. We also inferred the potential candidate regulators and their associated target sets in HCC using the importance values output by the random forest model. We observed a large overlap between our predicted target sets and targets identified using transfection or knockdown experiments confirming the accuracy of our model. An interesting future step would be to distinguish between upregulated and downregulated targets. Lastly, we found that one of our predicted candidate regulators, E2F6, is predictive of survival time in HCC patients. To the best of our knowledge, this is the first integrative model of multidimensional TCGA data for HCC. Our results will be instrumental in further studies of dysregulated gene expression control mechanisms in HCC.

## Figures and Tables

**Table 1:** List of candidate regulators.

Regulator	Avg. rank	log⁡FC	*p* value
Methylation	86	—	—
CNV	171	—	—
GLIS3	195	−0.61	0.008
TCF3	195	0.81	1.95*e* − 13
HIC2	199	0.48	3*e* − 03
ZEB1	205	−0.06	0.629
SPIB	206	−1.59	0.0019
GLIS2	208	0.52	0.05
TCF4	209	0.03	0.881
REST	209	−0.57	0.029
PKNOX2	212	−1.12	0.00017
GLI2	213	−1.33	0.0001
NFIA	214	−0.69	2.63*e* − 06
ID4	214	−1.24	5.69*e* − 05
MEF2A	215	−0.36	0.002
E2F6	216	0.15	0.187
ELF4	218	−0.25	0.28
ZBTB7C	223	−0.92	0.0002
TEAD4	223	0.62	0.02
PRDM1	224	−0.29	0.183
MEIS3	225	0.05	0.89
NR2F1	226	−0.82	0.0004
ZNF143	228	0.47	4.58*e* − 07
miR.766.3p	283	0.60	0.002
miR.335.3p	283	−0.17	0.39
miR.122.5p	284	−0.50	0.083

**Table 2:** Analysis of predicted target sets for candidate regulators.

Regulator	Target overlap	Enriched GO terms	Enrich. *p* value
TCF3	140	Cell cycle process	3.7*e* − 08
ZEB1	126	—	—
SPIB	146	Immune system process	7*e* − 13
TCF4	138	Extracellular matrix organization	2.1*e* − 03
REST	143	—	—
MEF2A	155	—	—
E2F6	165	Cell cycle process	3.5*e* − 22
PRDM1	142	Immune system process	4.5*e* − 29
NR2F1	135	—	—
ZNF143	158	Chromosome segregation	4*e* − 03
miR.122.5p	169	—	—
